# Mechanisms of lipid oxidation in water‐in‐oil emulsions and oxidomics‐guided discovery of targeted protective approaches

**DOI:** 10.1111/1541-4337.13158

**Published:** 2023-04-25

**Authors:** Yifan Bao, Marc Pignitter

**Affiliations:** ^1^ Institute of Physiological Chemistry Faculty of Chemistry, University of Vienna Vienna Austria; ^2^ Vienna Doctoral School in Chemistry (DoSChem) University of Vienna Vienna Austria

**Keywords:** interface, lipid oxidation, oxidomics, W/O emulsion, water droplets

## Abstract

Lipid oxidation is an inevitable event during the processing, storage, and even consumption of lipid‐containing food, which may cause adverse effects on both food quality and human health. Water‐in‐oil (W/O) food emulsions contain a high content of lipids and small water droplets, which renders them vulnerable to lipid oxidation. The present review provides comprehensive insights into the lipid oxidation of W/O food emulsions. The key influential factors of lipid oxidation in W/O food emulsions are presented systematically. To better interpret the specific mechanisms of lipid oxidation in W/O food emulsions, a comprehensive detection method, oxidative lipidomics (oxidomics), is proposed to identify novel markers, which not only tracks the chemical molecules but also considers the changes in supramolecular properties, sensory properties, and nutritional value. The microstructure of emulsions, components from both phases, emulsifiers, pH, temperature, and light should be taken into account to identify specific oxidation markers. A correlation of these novel oxidation markers with the shelf life, the organoleptic properties, and the nutritional value of W/O food emulsions should be applied to develop targeted protective approaches for limiting lipid oxidation. Accordingly, the processing parameters, the application of antioxidants and emulsifiers, as well as packing and storage conditions can be optimized to develop W/O emulsions with improved oxidative stability. This review may help in emphasizing the future research priorities of investigating the mechanisms of lipid oxidation in W/O emulsion by oxidomics, leading to practical solutions for the food industry to prevent oxidative rancidity in W/O food emulsions.

**Abbreviations**
AMVN: 2,2′‐azobis(2,4‐dimethylvaleronitrile)
BHT: butylated hydroxytoluene
CMC: critical micelle concentration
DAG: diacylglycerols
DMPO: 5,5‐dimethyl‐1‐pyrroline *N*‐oxide
DOPC: 1,2‐dioleoyl‐*sn*‐glycero‐3‐phosphocholine
DTAB: cationic dodecyltrimethylammonium bromide
EPR: electron paramagnetic resonance
FFA: free fatty acids
HLB: hydrophilic–lipophilic balance
MAG: monoacylglycerols
PC: phosphatidylcholine
PE: phosphatidylethanolamine
PGPR: polyglycerol polyricinoleate
PI: phosphatidylinositol
PUFA: polyunsaturated fatty acids
SDS: anionic sodium dodecyl sulfate
TAG: triacylglycerols
Tween 20: nonionic polyoxyethylene sorbitan monolaurate
UFA: unsaturated fatty acids
W/O: water‐in‐oil
WPI: whey protein isolate

## 1 INTRODUCTION

A water‐in‐oil (W/O) emulsion consists of water droplets dispersed in a lipid continuous phase, stabilized by small molecular surfactants, emulsifiers, or particles by decreasing the tension between the oil and water interface (Chen, Qiu, et al., 2016). In the food industry, W/O emulsions represent the basis of various food products, such as margarine, butter, chocolate, veiled extra olive oils, fat replacer, whipped cream, and ice cream (Ghosh & Rousseau, 2011; Pandolsook & Kupongsak, 2019; Zembyla et al., 2020). In addition, W/O emulsions can encapsulate, protect, and deliver some hydrophilic bioactive compounds (such as minerals, iron, peptides, and proteins) in water droplets dispersed in oil‐based food systems, thus acting as a sustained release system for these bioactive agents (Nadin et al., 2014; Prichapan et al., 2018; Zhu et al., 2016). However, because of the hydrophilic and hydrophobic compounds in emulsified foods, which may react with each other, the lipid oxidation in the food emulsions is more complex than in crude oils (Ushikubo & Cunha, 2014). Lipid oxidation might not only be affected by chemical factors, such as the fatty acid composition, transition metals (Laguerre, Bily, et al., 2020), lipase, and lipoxygenase (Lampi et al., 2020) but also by the physical characteristics of an emulsion. Specifically, the major site of lipid oxidation is the water–oil interface, and it can be proceeded readily by an unstable physical structure. A considerable body of research has found that the molecular structure of emulsifiers (Chen, Rao, et al., 2016; Chen, Qiu, et al., 2016), water contents (Kim et al., 2015), and emulsification equipment and methods (Lignel et al., 2017; Raviadaran et al., 2019) have a substantial influence on the physicochemical stability of W/O emulsions. Moreover, the stability of W/O emulsions during long‐term storage is not only affected by intrinsic properties but also by extrinsic factors such as temperature and light (Mohamad et al., 2019). In addition, the oxidative deterioration of W/O emulsions persists in the digestion process after the consumption by consumers. Lipid oxidation in W/O food emulsions may accompany the food supply chain until reaching consumers, causing irreversible untoward effects.

The oxidation of lipids produces free radicals, which not only can accelerate the rate of lipid oxidation but also lead to the loss of sensory quality and nutritional components, generation of toxic compounds, food degradation, and limited shelf life of lipid‐containing foods and may eventually jeopardize consumers’ health (Clarke et al., 2020; Serini et al., 2011). Since lipid oxidation in W/O emulsions affects commercial and nutritional value, discussing these complex oxidation processes theoretically and approving protective approaches are of great significance to both food industries and customers.

However, despite decades of research on lipid oxidation in W/O food emulsions, limited attention has been received in previous review studies on lipid oxidation in such emulsions. The available reviews have provided only brief summaries regarding lipid oxidation in W/O emulsions, as mentioned in reviews about the lipid oxidation in various types of emulsions (Coupland & McClements, 1996) or low‐moisture food (Barden & Decker, 2016). Some focused on a limited aspect, such as the lipid oxidation in specific W/O food emulsions like bulk oils (Budilarto & Kamal‐Eldin, 2015), the antioxidant (Laguerre et al., 2015), or interfacial behaviors of emulsifiers (Zembyla et al., 2020), such as phospholipids (Pichot et al., 2013). Despite a lot of efforts, the reactions during the lipid oxidation in W/O emulsions remain unpredictable due to multiple influencing factors, the consequences of lipid oxidation in W/O emulsions are still not clear enough, and the methods to prevent lipid oxidation in W/O emulsions still need to be improved. In light of this, the review covers various aspects of the topic, including the factors affecting lipid oxidation, the mechanisms of lipid oxidation, and the strategies for preventing lipid oxidation in W/O food emulsions. Additionally, the review will also highlight recent advancements in the field and provide insights into future directions of research. By focusing on the newest findings in this field, this review suggests a novel approach termed oxidomics to build up a holistic view of the network of chemical changes, physical properties, sensory properties, and nutritional values and precisely track the markers of the lipid oxidation in W/O food emulsions. Thus, applying oxidomics facilitates developing strategies to prevent lipid oxidation of W/O emulsions. The current review translates the fundamentals of lipid oxidation mechanisms in W/O emulsions to preventive measures relevant to the food industry by considering a global view to evaluate the quality of W/O emulsions, enhance their oxidative stability, and improve their sensory characteristics and nutritional value. Overall, the current review will provide a valuable resource for researchers and professionals working in the area of lipid oxidation in W/O food emulsions.

## 2 FACTORS AFFECTING LIPID OXIDATION IN W/O EMULSIONS

As a complex food system, W/O emulsion consists of diverse classes of molecules, which might come into contact with each other under different conditions, thereby affecting the lipid oxidation process. In lipid oxidation chain reaction, components from both water and oil phases, such as highly polar lipid hydroperoxides, transition metals (Fox & Dulay, 1993), and minor lipid components, may affect the transfer of antioxidants between and within the phases (Ambrosone et al., 2007), thus influencing the lipid oxidation in W/O emulsion (Mosca et al., 2008a). Furthermore, the interfacial tension between the water and oil phase may also alter the location of prooxidants and antioxidants, thereby affecting the lipid oxidation in W/O emulsions as well (Huan et al., 2017; Mosca et al., 2010; Steenson & Min, 2000). Other exogenous physical characteristics such as light and temperature also have effects on lipid oxidation. A comprehensive understanding of the factors causing lipid oxidation is vital to improve the shelf life and maintain the nutritional value of W/O emulsions.

### 2.1 Intrinsic components of W/O emulsions

#### 2.1.1 Fatty acid composition

It has been well documented that the fatty acid composition of W/O emulsion is closely related to its oxidative stability. Considering the health benefits, the W/O food emulsions containing unsaturated fatty acids (UFA) are favored by consumers; however, the lipid oxidation in W/O emulsions can also be triggered by lipid radicals generated by UFA (Chaix et al., 2014; Dickinson, 2012). A positive correlation was found between the lipid oxidation rate in W/O emulsions and the amount of linolenic acid in the lipid phase (Fox & Dulay, 1993). Interesting phenomena were observed in W/O emulsions consisting of various oils. The highest oxidation rate was observed in an emulsion consisting of soybean oil, followed by sunflower oil, canola oil, and olive oil, which were actually not related to the contents of UFA but to polyunsaturated fatty acids (PUFA), specifically, linoleic acid and linolenic acid (Lee & Choe, 2013). Similar findings were observed in W/O emulsions based on sunflower oil, soybean oil, flaxseed oil, linseed oil, camelina oil, rapeseed oil, and olive oil (Dridi et al., 2016). As generally known, oxidative stability may also be affected by the position of the double bonds (Barden & Decker, 2016). Moreover, the position of the fatty acid at the glycerol may also affect oxidative stability, showing a high stability of *sn*‐2 fatty acid in triacylglycerols (TAG) (Yamamoto et al., 2014).

#### 2.1.2 Minor components

TAG are the major compounds of W/O emulsions, with minor compounds such as polar TAG polymers, monoacylglycerols (MAG), diacylglycerols (DAG), free fatty acids (FFA), phospholipids, sterols, tocopherols, polyphenols, pigments, or proteins like lipoxygenase. In this regard, the prooxidative or antioxidative activities of these minor compounds need to be considered, as well as their antagonistic or synergistic effects (Table 1). The effects of MAG and DAG, the predominant minor compounds in W/O emulsions, on lipid oxidation have been reported differently depending on the concentrations. The addition of 0−2.5 wt% DAG or MAG in the stripped soybean oil, a W/O emulsion, had no significant influence on the oxidative stability (Chen et al., 2014). A W/O emulsion based on corn oil, which contained 0.3% MAG and 5.1% DAG, was observed to be more prone to lipid oxidation than natural corn oil with undetected MAG and 1.4% DAG (Wang et al., 2005). However, lipid oxidation protective effects of 1−3 wt% MAG were observed in olive oil at 60°C (Gomes et al., 2010), which might be due to the emulsifying property of MAG. Likewise, as an emulsifier in water‐in‐perilla oil emulsion, MAG acted as an antioxidant, which can be attributed to the physical barrier it forms, leading to reduced oxygen diffusion and degradation of chlorophyll and polyphenols (Kim & Choe, 2012).

**TABLE 1:** The influence of minor compounds on lipid oxidation of W/O emulsions and possible mechanisms.

Components	Influence	Relating factors	Possible mechanisms
MAG and DAG	Anti/prooxidative	Concentration	Building physical barriers to decrease the oxygen diffusion
Free fatty acids	Prooxidative	Degree of unsaturation; chain length; conformational feature	Accelerating the oxidation of sterol and the transfer of oxygen into the lipid phase; generating prooxidative complexes with transition metals; form reverse micelles
Phospholipids	Antioxidative	Concentration	Chelating transition metals
	Prooxidative	Concentration	Decreasing the interfacial tension
Phytosterols	Antioxidative	Concentration	Inhibiting lipid oxidation by acting as hydrogen donators
	Prooxidative	Degradation	
Tocopherols	Antioxidative	Phospholipids	Reacting with free radicals
	Prooxidative	High concentrations; high water content	Oxidized tocopherols act as prooxidants;
Polyphenols	Antioxidative	Concentration	Reacting with free radicals; metal chelator
Pigments	Antioxidative	Concentration; dark; low oxygen content	Converting excited photosensitizers or singlet oxygen to less reactive states; scavenging free radicals
	Prooxidative	Light; thermal degradation; high oxygen content	Promoting the formation of singlet oxygen
Lipoxygenase	Prooxidative	Ferrous iron; hydroperoxides	Enzymatic oxidation

FFA, containing a hydrophobic tail and a hydrophilic head, can act as surface‐active compounds to accelerate the lipid oxidation in W/O emulsions by transferring the oxygen into the lipid phase, generating prooxidative complexes with transition metals, as well as forming reverse micelles (Chaiyasit et al., 2008; Chen, McClements, et al., 2011; Miyashita & Takagi, 1986). Even at an extremely low concentration, FFA can show strong prooxidant activity. For example, 0.05% (w/w) FFA in water‐in‐walnut oil emulsions significantly accelerated the lipid oxidation (Yi et al., 2013). In this study, the lipid oxidation rate was demonstrated to be closely related to the degree of unsaturation and the chain length of FFA. FFA with a higher degree of unsaturation had a stronger prooxidant effect, in the order of linolenic acid, linoleic acid, and oleic acid. A long chain length of saturated FFA promoted the lipid oxidation in W/O emulsions with the following order, lauric acid > palmitic acid > stearic acid. Besides the interaction between FFA and oxygen at the water–oil surface, the polarity of FFA renders the water–oil interface more negatively charged, thus transferring more transition metals to the interface area. Moreover, the bow shape of *cis*‐UFA may lead to a stronger attraction to active oxygen. The addition of oleic acid (18:1, *cis*) and linoleic acid (18:2, *cis*–*cis*) in water‐in‐walnut oil emulsion showed stronger prooxidant activity than their geometric isomers, elaidic acid (18:1, *trans*) and linoleic acid (18:2, *trans*–*trans*), respectively (Yi et al., 2013). In addition, FFA can also accelerate the oxidation of sterols. Cholesterol and β‐sitosterol oxidized more rapidly in the presence of 5% stearic acid, oleic acid, linoleic acid, and α‐linolenic acid (Xu et al., 2011).

Phospholipids are a good source of antioxidants in W/O emulsion due to the transition metals‐chelating ability of the anionic phosphate head group (Yang et al., 2019) and their capability to regenerate antioxidants such as α‐tocopherol or other phenols (Pokorný & Korczak, 2001). However, the reverse micelles or lamellar structure formed by phospholipids might switch the location of hydrophilic and amphiphilic prooxidants (Chaiyasit et al., 2007; Cui & Decker, 2016; Cui et al., 2014), which may ultimately facilitate lipid oxidation.

There are also trace amounts of antioxidants in W/O emulsions capable to prevent lipid oxidation. Polyphenols prevent lipid oxidation in bulk oils by scavenging free radicals at the water–oil interface (Budilarto & Kamal‐Eldin, 2015). Phytosterols are particularly prone to oxidation due to their surface‐active property. They inhibit the initial oxidation by acting as a hydrogen donator. A nuclear magnetic resonance study monitored the oxidative status of margarine with different concentrations of phytosterols subjected to 180°C for 240 min. The oxidation‐delaying effects of phytosterols were dose dependent, which demonstrated the antioxidant activity of phytosterols. Interestingly, another study, which followed the fate of phytosterols in margarine during storage, found that the decreasing amount of sterol did not correspond to the increasing amount of sterol oxidation products (Rudzińska et al., 2014). Considering the free radical mechanism of the phytosterol degradation, this phenomenon leaves the question of whether sterol oxidation products are involved in lipid oxidation reactions, thereby weakening the oxidative stability of W/O emulsions. Similarly, tocopherols are the common antioxidants in plant‐based W/O emulsions with the antioxidant activity of δ‐tocopherol > γ‐tocopherol > β‐tocopherol > α‐tocopherol (Chaiyasit et al., 2007). They are located in the oil phase and delay lipid oxidation by reacting with free radicals. Furthermore, the oxidized tocopherols may in turn promote lipid oxidation (Kim & Min, 2008). A high content of water may also promote the decomposition of α‐tocopherol (Kim et al., 2015).

Chlorophyll and carotenoids, two major pigments in W/O emulsion made from vegetable oils, sustain the photooxidation. As a photosensitizer, chlorophyll promotes the formation of singlet oxygen in the presence of light (Laguerre, Tenon, et al., 2020). More surprisingly, chlorophyll and its degradation product pheophytin can protect against lipid oxidation in dark by scavenging peroxyl and other free radicals (Endo et al., 1985). Carotenoids may inhibit the lipid oxidation caused by scavenging singlet oxygen and excited photosensitizers, in a low‐oxygen environment. However, carotenoid degradation products induced by a high storage temperature (60°C) in dark were demonstrated to be prooxidants facilitating lipid oxidation (Steenson & Min, 2000).

W/O food emulsions are mainly based on plant oil or animal oil, indicating that enzymatic oxidation by endogenous enzymes is inevitable in W/O food emulsions. In the W/O food emulsions containing animal oil, the active site of lipoxygenases contains ferrous iron and generates a conjugated hydroperoxyl diene and alkyl radical by abstracting a hydrogen atom from highly unstable unsaturated lipids (Ghnimi et al., 2017). Hydroperoxide, in turn, converts ferric to ferrous iron, which further maintains the activity of lipoxygenase. Higher free acidity and rancidity were observed in filtered olive oil than in veiled virgin olive oil, which can be attributed to the removal of antioxidants from the water phase, thus decreasing the inhibiting effects of amphiphilic antioxidants on enzymatic oxidation (Kalogeropoulos & Tsimidou, 2014).

Even at low concentrations, minor compounds can still influence the lipid oxidation in W/O emulsions. The process of lipid oxidation can be affected by their chemical changes and the interactions between these minor compounds. Hence, more studies are required in the future to show the chemical and spatiotemporal fate of these minor compounds as well as the association between them in lipid oxidation.

#### 2.1.3 Transition metals

In general, the lipid phase is nonionizing or low‐ionizing (Wang, Yang, et al., 2021). However, the dispersed water droplets contain various ionic compounds (Mhatre et al., 2015), especially transition metals. As food fortifiers (Shubham et al., 2020) or physical stabilizers to reduce Ostwald ripening (Márquez et al., 2010; Nadin et al., 2014; Zhu et al., 2016), these transition metals also catalyze lipid oxidation more efficiently than temperature or light by increasing the electron transfer (Chaijan & Panpipat, 2016). Transition metals, both in water and oil phases, such as iron, magnesium, and copper, can rapidly diffuse into the water–oil interface due to their hydrophilicity. By catalyzing the generation of free radicals, such as hydroxyl and peroxyl radicals, these transition metals initiate the chain reaction, thus accelerating the lipid oxidation in W/O emulsions (Guzun‐Cojocaru et al., 2010; Waraho et al., 2011). The fundamental reactions of transition metals promoting lipid oxidation in W/O emulsions are as follows (Choe & Min, 2006):

$$M^{n+}+\mathrm{RH}\to M^{(n-1)+}+R\cdot +H^{+}$$

$$\mathrm{ROOH}+M^{(n-1)+}\to \mathrm{RO}\cdot +M^{n+}+{\mathrm{OH}}^{-}$$

$$\mathrm{ROOH}+M^{n+}\to \mathrm{ROO}\cdot +M^{(n-1)+}+H^{+}$$

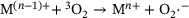




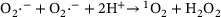




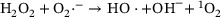




Accordingly, the prooxidative effects of transition metals can be accelerated when the W/O emulsions contain significant amounts of UFA, or by enhancing the diffusion of oxygen (Ghnimi et al., 2017). In addition, low pH or the presence of ascorbic acid can provide a higher solubility for transition metals, thereby promoting the lipid oxidation in W/O emulsions (Choe & Min, 2006; Dridi et al., 2016). The oxidation rates in different W/O emulsions based on rapeseed oil, linseed oil, camelina oil, and olive oil were consistent with the concentration of ferrous sulfate in the water phase (Dridi et al., 2016). However, it is interesting to note that in this study the lipid oxidation rate in refined rapeseed‐based W/O emulsions with differently enriched ferrous solutions showed the following order: ferrous chloride > ferrous sulfate > ferrous lactate > ferrous gluconate. The prooxidant activity of transition metals can be inhibited by their counter ions with metal chelating ability, such as lactate and gluconate. Furthermore, the reaction between lipid compounds and transition metals can be retarded by adjusting the charge state, thickness, tension, as well as density of the water–oil interface (Berendsen et al., 2014).

#### 2.1.4 pH

The pH can play an important role in lipid oxidation in W/O food emulsions. In general, a low‐pH environment is favorable for lipid oxidation, while high pH can reduce the rate of lipid oxidation. The pH of the W/O emulsion also affects the stability of the microstructure (Pichot et al., 2013), thus influencing the solubility of oxygen, which can influence the rate of lipid oxidation. This might be further explained by the interfacial behaviors of emulsifiers influenced by pH. For instance, W/O emulsions prepared with whey protein isolate (WPI) at pH 7.0 are less prone to lipid oxidation than at pH 3.0 (Yi et al., 2014). In addition, the activity of lipoxygenase or antioxidants can also be influenced by pH, thus affecting lipid oxidation in W/O emulsions. pH may mainly affect lipid oxidation via other factors, rather than acting as a direct determinant. Further research is needed to infer the direct or indirect role of pH in lipid oxidation in W/O food emulsions.

### 2.2 Microstructure of W/O emulsion

**FIGURE 1:**
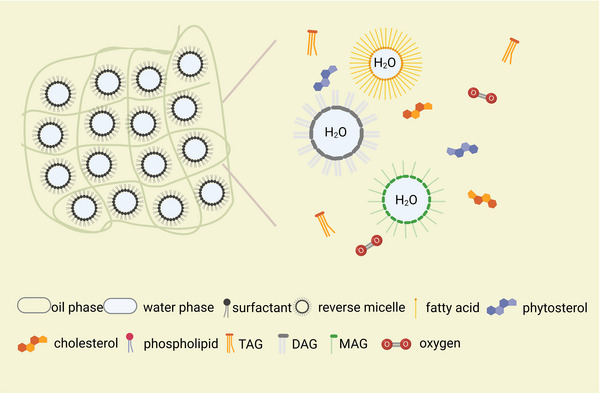
Microstructures of water‐in‐oil (W/O) emulsions and fat crystal network and reverse micelle formed by MAG, DAG, and FFA. MAG, monoacylglycerols; DAG, diacylglycerols; FFA, free fatty acids. Created with BioRender.com.

Given the thermodynamical instability of W/O food emulsions, the lipid oxidation process depends on the synergistic effects of both chemical and physical reactions. More specifically, W/O emulsions are stabilized by a fat crystal network, which encases the water phase in the oil phase by avoiding droplet coalescence (Figure 1). While the area between water and oil phases determines the dispersion of prooxidants and antioxidants in W/O emulsion systems, especially the distribution of initiator peroxyl radicals and the chemical reactions between these prooxidants or antioxidants and lipid compounds occur mainly at the water–oil interface (Mosca et al., 2010). Therefore, water concentration and the size and distribution of water droplets can be the decisive factors in lipid oxidation rate, since they directly determine the interfacial phenomena of emulsions (Ambrosone, Mosca, et al., 2006; Berton‐Carabin et al., 2014; Mosca et al., 2008b). Furthermore, emulsifiers can modify the interfacial tension in W/O food emulsions, thereby affecting the microstructure and acting as an important regulating factor on the lipid oxidation (Calligaris et al., 2004; Mosca et al., 2010; Waraho et al., 2011).

#### 2.2.1 Colloidal structures in W/O emulsions

As a heterogeneous system, W/O emulsions rich in amphiphilic or hydrophilic compounds often form colloidal structures. The interface between colloidal structures and the oil phase allows the copresence of both hydrophilic and lipophilic compounds, and provides the environment for lipid oxidation, thus directly affecting oxidative stability (Xenakis et al., 2010).

Reverse micelles can be easily self‐assembled by amphiphilic hydroperoxides and some other surface‐active compounds such as MAG (hydrophilic–lipophilic balance [HLB] = 3.4–3.8), DAG (HLB = 1.8), sterols, FFA (HLB = 1.0), and phospholipids (HLB = 8.0) (Dalby & Care, 1999; Moulik, 1996), which can be mainly characterized by their HLB (Matsaridou et al., 2012). The monolayer of the reverse micelle is formed by surface‐active compounds, which trap the water and hydrophilic components in a nanoscale “water pool” (Figure 1).

**FIGURE 2:**
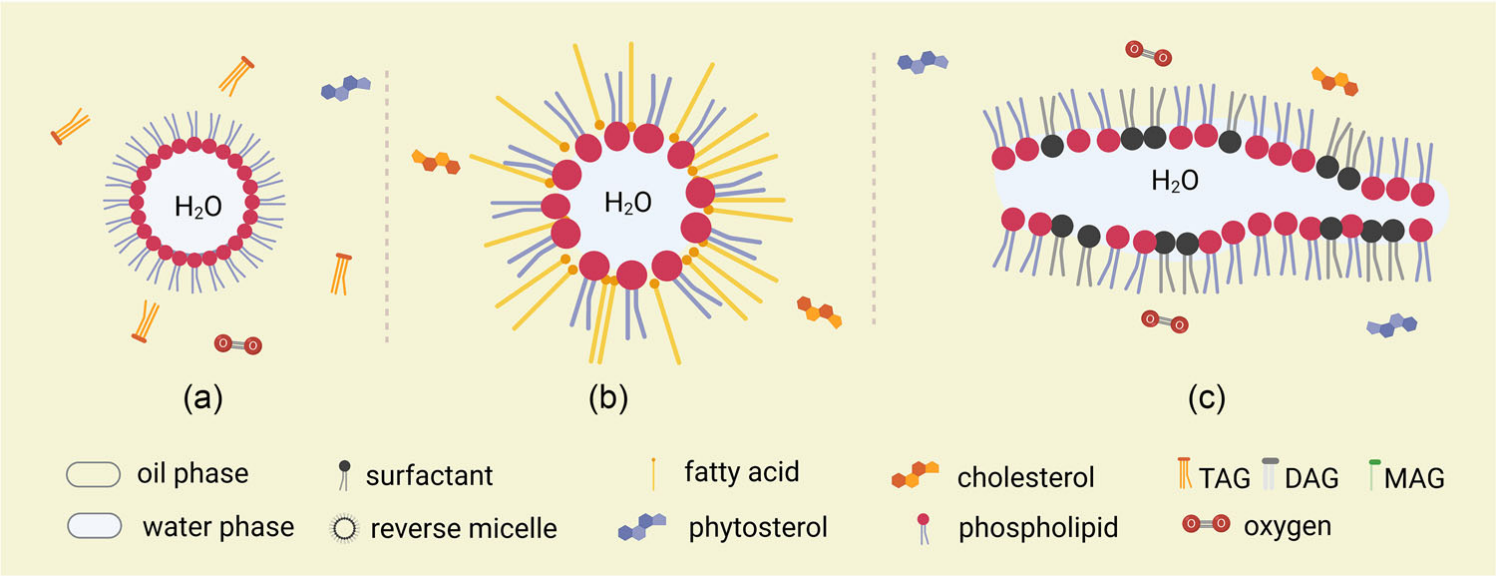
Colloidal structures formed by small molecules. (a) Reverse micelles formed by phospholipids. (b) Reverse micelles formed by FFA and phospholipids. (c) Lamellar structure. FFA, free fatty acids. Created with BioRender.com.

Due to the Van der Waals attraction, the size of larger water droplets increases gradually by merging with smaller water droplets (Ostwald ripening) (Verma et al., 2011). More specifically, the movement of reverse micelles follows Brownian motion and allows the exchange of the water content in the “water pool” (Luisi & Straub, 1984), thereby altering the surface and affecting the lipid oxidation rate. Phospholipids (e.g., phosphatidylcholine [PC], phosphatidylethanolamine [PE], phosphatidylinositol [PI]) form sphere reverse micelles in natural oils with lower water content (Figure 2a). PC has been demonstrated to form a more compatible monolayer than PE and PI, thus enhancing the stability of micelles (Handa et al., 1990). Besides forming the layer of the micelles, PE prevents iron‐catalyzed oxidation by donating a hydrogen atom (Choe & Choe, 2016) and PI inhibits the chlorophyll‐photosensitized oxidation (Choe et al., 2014). Moreover, various reverse micelle structures can be formed from phospholipids in W/O emulsions, depending on the concentrations of phospholipids, pH, temperature, and the existence of other surfactants (Pichot et al., 2013). For example, FFA, besides acting as prooxidant, also contribute to building up the colloidal structure in W/O emulsion as a cosurfactant (Figure 2b). A minimal change in the water content and the concentration of surface‐active compounds was demonstrated to influence the size and shape of reverse micelles (Lehtinen et al., 2017). In a study about stripped corn oil, oleic acid was found to co‐assemble with 1,2‐dioleoyl‐*sn*‐glycero‐3‐phosphocholine (DOPC) to form reverse micelles and decrease the pH in the core region of reverse micelles, thus increasing the lipid oxidation rate (Kittipongpittaya et al., 2014). The addition of oleic acid (0–100 mmol/kg oil) decreased the size of reverse micelles in stripped corn oil, whereas the addition of PC (0–1 mmol/kg oil) increased it (Chaiyasit et al., 2008). In the presence of phospholipid and some other surfactants, an inverse lamellar structure (Figure 2c) might also be formed (Chaiyasit et al., 2007). In W/O emulsions based on rapeseed oil, the addition of oleic acid (0, 5, 10, or 20 wt%) was demonstrated to have different effects on the lecithin (1 wt%) micellar structure formation depending on the water content (Penttila et al., 2019). A promoting effect of oleic acid on the formation of lecithin reverse sphere micelles was observed at a very low water concentration (0.019 wt%), and the size of reverse sphere micelles increased by about 15% with 0.07 wt% water addition. With the addition of 10 and 20 wt% of oleic acid, acting as a cosurfactant, the “water pool” of reverse micelles was expanded and merged to form a lamellar structure. Hence, the promoting effects of cosurfactants such as FFA and the inhibiting effects of phospholipids on lipid oxidation in W/O emulsions can not only be explained by chemical reactions but might also be attributed to their ability to modify the size and shape of reverse micelles, probably through Brownian motion.

**FIGURE 3:**
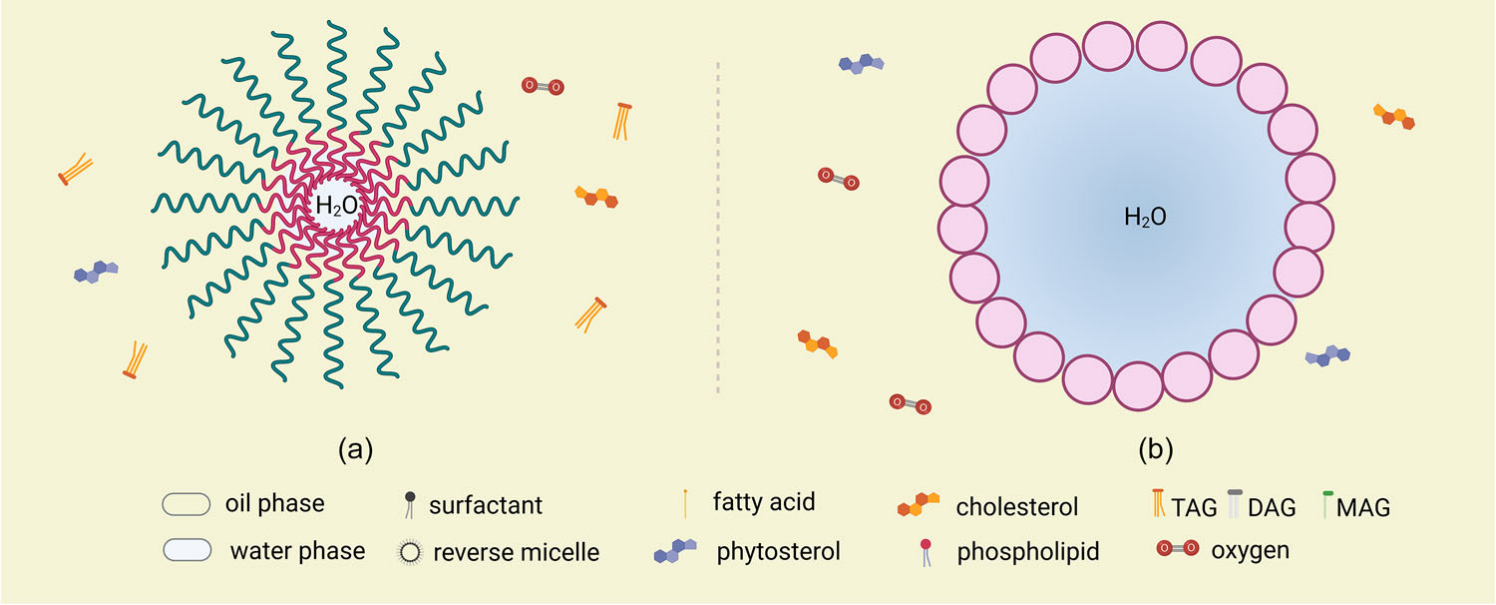
Colloidal structures formed by (a) biopolymers and (b) particles. Created with BioRender.com.

Besides micelles formed by small molecules, many biopolymers with amino acid or sugar groups are applied in the food industry due to their role as both antioxidants and emulsifiers to delay the lipid oxidation in W/O emulsions by interacting with free radicals directly or inhibiting the diffusion of prooxidants and oxygen (Coupland & McClements, 1996). By thickening the monolayers of colloids, W/O emulsions stabilized by biopolymers have a significantly higher physical stability (Figure 3a). For example, emulsions stabilized by octenyl succinate starch showed a delay of lipid oxidation and a better physical stability because the barrier of the interface between water and lipid phase was strengthened by the starch, thus the Ostwald ripening was delayed (Wang et al., 2017). Furthermore, proteins can promote the physical and oxidative stability of W/O emulsions due to their excellent antioxidant activity, stability, and emulsifying property (Kerasioti et al., 2016; Zhao et al., 2018). WPI has been confirmed as an emulsifier to chelate the transition metals and as a free radical scavenger, thus retarding lipid oxidation (Khan et al., 2019).

In addition, to form a solid shell around the water droplet, “Pickering stabilization” uses insoluble solid particles to form the colloid structure in the W/O emulsions (Pickering, 1907). Generally, these stabilizers, such as silica, cellulose, lignin, starch, and polyphenol crystals, are nature‐ or bioderived with the size of nanoscale or even microscale (Zembyla et al., 2020). Due to the larger size (10 nm to 100 μm) than other surfactants (1–50 nm) (Xiao et al., 2016), particles can form micelles with a larger water pool and a thicker monolayer, thus preventing lipid oxidation at the interface (Figure 3b). It has been proposed to develop Pickering W/O emulsions with thicker monolayers to inhibit lipid oxidation. However, the lipid oxidation‐preventing effects of Pickering W/O emulsions seem to be limited, since particles tend to reach a critical thickness and did not show higher oxidative stability with increasing particle sizes (Schröder et al., 2019).

#### 2.2.2 The role of emulsifiers on interfacial oxidation

As the stabilizers of the W/O emulsion, emulsifiers act as thickening agents to prevent the water droplets from sedimentation (Borreani et al., 2019). The location and reactivity of prooxidative transition metals, lipid hydroperoxides, antioxidants, and metal chelators can be affected by emulsifiers (Mosca et al., 2010; Waraho et al., 2011). Hence, emulsifiers have a substantial controlling effect on lipid oxidation in the W/O emulsion system by regulating interfacial behavior between the aqueous and oil phases.

The lipid oxidation‐preventing effects of emulsifiers can be influenced by various factors, such as the charge state and the concentration. Generally, uncharged and lipophilic emulsifiers, such as Span surfactants, are responsible for the reduction of interfacial tension, thus promoting the diffusion of antioxidants into the interfacial region (Opawale & Burgess, 1998; Real et al., 2021). On the other hand, electrically charged emulsifiers (e.g., proteins, phospholipids, anionic or cationic surfactants) can transfer the charge to the micelles thereby affecting the lipid oxidation chain reaction (Shahidi & Zhong, 2011). In a study about the influence of emulsifiers on lipid oxidation in water‐in‐walnut oil emulsions, lipophilic polyglycerol polyricinoleate (PGPR), cationic dodecyltrimethylammonium bromide (DTAB), anionic sodium dodecyl sulfate (SDS), and nonionic polyoxyethylene sorbitan monolaurate (Tween 20) were used as stabilizers (Yi et al., 2014). A trend of lipid oxidation rate was recorded as PGPR + DTAB > PGPR ≈ PGPR + Tween 20 > PGPR + SDS. SDS can form negatively charged micelles, which bind to transition metals thereby decreasing the concentration of prooxidants at the water–oil interface. So, the anionic emulsifiers interrupt the free radical oxidation chain reaction, while the cationic emulsifiers might stimulate the reaction in W/O emulsions.

It is also worth mentioning that emulsifiers can affect the efficacy of antioxidants. The addition of α‐tocopherol prevented the degradation of PC (Lee & Choe, 2013), and PC in turn enhanced the antioxidant activity of α‐tocopherol (Koga & Terao, 1995), suggesting that emulsifiers may mediate the chemical reaction of antioxidants and prooxidants by facilitating the micelle transfer. However, the addition of 2.5 wt% MAG inhibited the antioxidant activity of α‐tocopherol (40 μM) in stripped soybean oil. The interfacial tension was also decreased from 17.8 to 1.1 mN/m, which indicates that the movement of MAG to the surface of the oil resulted in the faster consumption of α‐tocopherol (Chen et al., 2014).

#### 2.2.3 The oxidant–antioxidant phenomena at the interfacial and surface area

**FIGURE 4:**
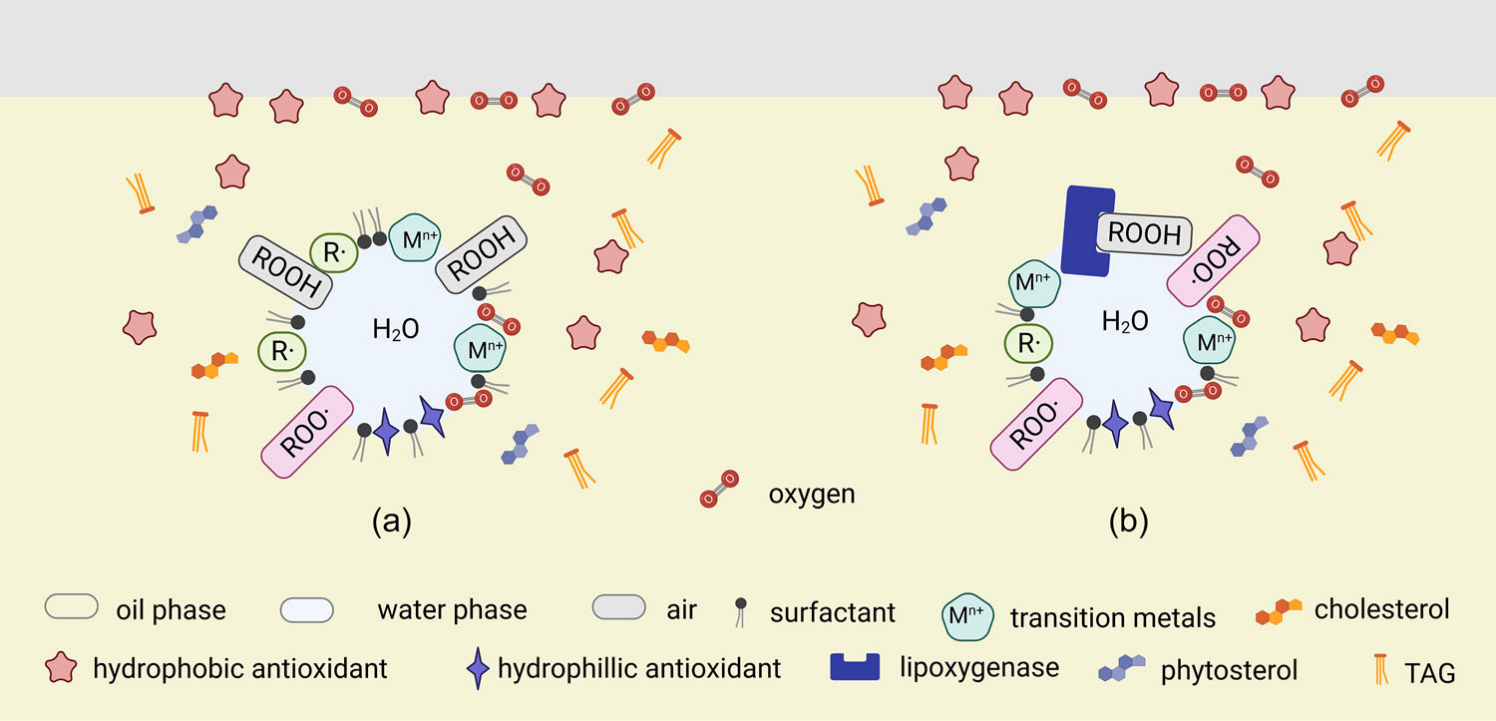
The oxidant–antioxidant behaviors in water‐in‐oil (W/O) emulsions. (a) The distribution of antioxidants in W/O emulsions (b) The contact region of lipoxygenase. Created with BioRender.com.

Interestingly, in W/O emulsions, the polar antioxidants show enhanced activity in a low‐polar environment (Budilarto & Kamal‐Eldin, 2015). This antioxidant–prooxidant balance seems to point to a polar paradox theory (Shahidi & Zhong, 2011). The hydrophilic antioxidants aggregate at the water–oil interface, while the hydrophobic antioxidants migrate to the air–oil interface (Figure 4a). For example, in the stripped soybean oil with reverse micelles formed by DOPC, a better antioxidant activity was observed after the addition of hydrophilic Trolox instead of lipophilic α‐tocopherol (Chen, Han, et al., 2011). In addition, the microstructure formed by triglycerides and amphiphilic and hydrophilic compounds also appears as a privileged contact region for lipoxygenase (Xenakis et al., 2010) (Figure 4b).

Polar paradox theory explained the different interfacial phenomena of antioxidants with hydrophilic or hydrophobic properties (Farooq et al., 2021). However, such a theory cannot specify the antioxidative efficacy by the empirical term “polarity,” especially in a nonideal complex W/O emulsion system that contains various antioxidants. Hence, a new series of molecules, so called phenolipids, that are defined as phenolic compounds conjugated to a lipid moiety have been suggested to be used to reevaluate the relationship between antioxidant activity and polarity. Despite the validation and confirmation of the polar paradox theory by some studies using phenolipids (Kikuzaki et al., 2002; Mateos et al., 2015; Pereira‐Caro et al., 2009), the antioxidative efficacy of phenolipids in W/O emulsion foods was not always compatible with the polar paradox theory (Laguerre et al., 2011; Lopez‐Giraldo et al., 2009). For example, higher oxidative stability was observed in the olive oil‐based W/O emulsions enriched with phenolipids derived from homoprotocatechuic acid, dihydrocaffeic acid, caffeic acid, dihydroferulic acid, ferulic acid, and protocatechuic acid, than the emulsions enriched with phenolic compounds (Torres de Pinedo et al., 2007). A possible explanation of these observations, which are in contradiction to the polar paradox theory, is the interaction between free phenolic and metals bound to the anionic phospholipids, leading to the solubilization of the metals into the water phase, thus boosting the prooxidative effects of metals. Phenolipids, with the more hydrophobic property, fail to be located in the water phase (Laguerre et al., 2015). However, among phenolipids derived from the same phenolic antioxidants, the antioxidative activity has a nonlinear relation with hydrophobicity, which is also known as the cutoff effect of the polarity. The antioxidant activity of phenolipid increases with the chain length and decreases after reaching a critical chain length (Laguerre et al., 2013; Panya et al., 2010). The phenolipids with short or medium chain length enhanced the antioxidative activity of phenolic antioxidants in W/O emulsion, more than the ones with long chain length (Laguerre et al., 2009, 2010). These paradoxical cutoff effects might be related to the molecular size of phenolipids. The larger molecular size of phenolipids decreases their mobility in the W/O emulsions and leads to the aggregation of phenolipids in the oil phase, rather than functioning in the interfacial region (Shahidi & Zhong, 2011).

In fact, part of the antioxidative effects of phenolipids can still be explained by the polar paradox theory; therefore, both antioxidant and oxidant phenomena exist in W/O emulsion. A study investigated the concentration‐dependent effect of phenolic antioxidants and their phenolipids on their antioxidative activity in W/O emulsions (Zhong & Shahidi, 2012). The antioxidative effect showed a parabolic relation with concentration, rather than a linear relation. The critical concentration, which can be determined from the point of intersection of two parabolas, varied in different phenolic antioxidants and their phenolipids. It was suggested by Laguerre et al. (2017) that the mass transport of antioxidants may occur by diffusion, collision–exchange–separation, and micelle‐assisted transfer in oil‐in‐water emulsions, depending on their polarity and concentration. Similar mechanisms could potentially apply to W/O emulsions as well, which need to be further explored using novel techniques or novel methods based on traditional techniques.

Hence, the antioxidant–oxidant phenomenon cannot be simply explained by the polar paradox, the cutoff effect based on the polarity of phenolipids, or the mass transport phenomena. More systematic and nuanced theories considering all the intrinsic components, the microstructure, and also the exogenous factors need to be proposed to better interpret lipid oxidation inhibition in W/O emulsions and to possibly overcome the natural limitation of applying antioxidants with various polarities in W/O emulsions.

### 2.3 Exogenous prooxidative factors

It is difficult to interpret the effects of intrinsic components and colloidal structures on lipid oxidation in W/O emulsions, despite considering both chemical and physical parameters, such as the fatty acid composition, shape and size of the colloids, the charged state of micelles, and the interfacial tension. During the processing and storage of W/O food emulsions, exogenous prooxidative factors, such as oxygen content, light, and temperature, also influence lipid oxidation continuously.

#### 2.3.1 Oxygen

Oxygen is essential for the generation of hydroperoxide to propagate lipid oxidation. It has been confirmed that the lipid oxidation rates in processed W/O emulsions are much higher than in the natural W/O emulsions, such as bulk oils, which may be due to incorporating oxygen during the processing or packaging of emulsions (Dridi et al., 2016). Another study reported that W/O emulsion prepared by shaking for 15 min was more resistant to lipid oxidation than the emulsions shook for 45 min, which suggested that the area of the interface being exposed to oxygen is closely related to the preparation time of W/O emulsions (Ambrosone, Cinelli, et al., 2006).

#### 2.3.2 Light

W/O food emulsions are often sold in transparent containers; however, lipids are prone to photooxidation. Light might stimulate the generation of singlet oxygen by the energy transfer from a photosensitizer (e.g., chlorophyll, carotenoids, riboflavin), thus leading to the acceleration of lipid oxidation in W/O emulsions (Laguerre, Tenon, et al., 2020). Lipid oxidation in W/O emulsions can be promoted by light, especially in the shorter wavelengths (Sattar et al., 1976), but the oxidation caused by light can be less pronounced than the lipid oxidation induced by elevated temperatures (Kim & Choe, 2012).

#### 2.3.3 Temperature

Emulsions are susceptible to degradation under high temperatures (Farhoosh, 2021), which may not only be attributed to physical instability but also to accelerated lipid oxidation. Despite being stored at subzero temperatures, some W/O food emulsions still suffer from lipid oxidation, which may be attributed to the crystallization being more prone to happen in the water phase than the lipid phase, as well as the increase of viscosity (Calligaris et al., 2004, 2007).

The significant amount of work on the multiple factors causing lipid oxidation in W/O emulsions has drawn attention to the fact that the complex mechanisms need further exploration, both from physical and chemical dimensions. Nevertheless, it is difficult to make a breakthrough in monitoring the physical migration and chemical interaction of antioxidants and prooxidants at the interface with the commonly used approaches in this field.

## 3 MECHANISMS AND MARKERS OF LIPID OXIDATION IN W/O EMULSIONS

The mechanisms of lipid oxidation in W/O emulsions are similar to those in crude oils based on the free radical chain reaction (Choe & Min, 2006). During this process, all the generated radicals can act as catalyzers in the reaction, especially in the propagation stage (Laguerre, Tenon, et al., 2020). However, due to the presence of water in the emulsion system, lipid oxidation in W/O emulsions can be more complex. It is well agreed that the lipid oxidation of W/O emulsions is mainly occurring at the interface of lipid and aqueous phases (Villeneuve et al., 2018), and is also affected by (1) the chemical conditions, such as the concentration of hydroperoxides, components of water droplets and lipid phases, emulsifiers, and surfactants, as well as (2) physical conditions, such as temperature, light, and water–oil interfacial tension. Although tremendous studies have investigated the mechanism of lipid oxidation, the molecular mechanisms considering both chemical and physical conditions remain puzzled, especially in complex W/O emulsion food systems. To better understand the mechanisms of lipid oxidation, systematic novel approaches have been developed to detect the lipid oxidation of W/O emulsions from both physical and chemical perspectives.

### 3.1 Kinetics of lipid oxidation in W/O emulsions

In the assessment of the oxidative stability of W/O food emulsions, lipid oxidation kinetics studies are particularly important references to evaluate the rate of free radical chain reactions (Table 2). Most studies monitored the oxidation pathway in emulsion systems by accelerating oxidation with high temperature to estimate the shelf life; however, the initial pathways might be overlooked due to the univariate thermal control. In this regard, the kinetics of lipid oxidation in W/O emulsions in the early stage can be studied in a temperature‐dependent manner—triggering the initiation of lipid oxidation at lower temperatures. For instance, to better mimic the household conditions, Grüneis et al. (2019) focused on the kinetics of lipid oxidation in margarine heated at 25, 80, and 180°C. A higher reaction rate was observed for epoxidation than hydroperoxidation. This tendency increased with temperature as well.

**TABLE 2:** Representative lipid oxidation kinetics study of water‐in‐oil (W/O) emulsions.

W/O emulsions	Stimulation conditions	Monitoring variables
Margarine	Household cooking conditions (Grüneis et al., 2019)	Peroxide value, oxidized triacylglycerols
Butter	Storage in grocery (Veberg et al., 2007)	Peroxide value, odor, flavor, volatiles
Emulsion based on olive oil	Presence of free radicals (Mosca et al., 2010)	Hydroperoxide concentration, droplet size

As the initiator of photooxidation, light and oxygen exposure promoted lipid oxidation in W/O food emulsions. A study monitored the fluorescent emissions of lipid oxidation products in butter stored under air or nitrogen at 4°C and exposed to light to mimic the storage conditions in grocery (Veberg et al., 2007). In the nitrogen‐protected butter sample, the primary mechanisms were type I photosensitized reactions, whereas both photooxidation type I and type II reactions occurred in air‐exposed butter samples. Moreover, rather than physical initiators, W/O emulsions are exposed to chemical initiators to be close to the process of lipid oxidation during shelf life, thus better understanding the lipid oxidation kinetics. In a study about the oxidative status in W/O emulsion formulated with olive oil, lipophilic 2,2′‐azobis(2,4‐dimethylvaleronitrile) (AMVN), an azo radical, was used to initiate the oxidation reaction and a fluorescence method was used to monitor the oxidation status (Mosca et al., 2010). The diffusion rate of low amounts of AMVN was slowed by the cage effects of the oil phase. This effect was retarded by higher water content and larger water–oil surface area. In addition, carotenoids degradation was found to have a linear relationship with the dissociation of AMVN. Thus, the application of lipophilic radicals in the kinetics study of lipid oxidation in W/O emulsion allows monitoring not only the lipid oxidation status but also the degradation of minor antioxidant compounds.

Although taking the most influential factors of the lipid oxidation into account, it is still challenging to describe the lipid oxidation mechanism in W/O emulsions as linearly dependent on time. The duration of the lag phase can be the determining factor in the shelf life of W/O food emulsions. Regrettably, investigations into detailed physical and chemical changes during the initial stage of oxidation are lacking due to the complexity of the homogeneous system. Future studies would be desired to illustrate the unknown mechanisms of the initial phase of lipid oxidation in W/O emulsions. The limitation might be investigating the initial changes by “traditional” methods, such as titration experiments and chromatography techniques. Regarding the fact that the initial stage is based on the free radical reactions, detecting the formation of free radicals prior to the generation of hydroperoxides might be a promising approach to investigate the early stage of lipid oxidation. This hypothesis can be verified by the application of electron paramagnetic resonance (EPR), a sensitive technique to track the free radicals. 5,5‐Dimethyl‐1‐pyrroline *N*‐oxide (DMPO) was used as an electron spin trap in the kinetic study of the lipid oxidation of W/O emulsions based on rapeseed oil (Chen et al., 2017). Free radicals and aldehydes were mainly generated from oleic acid in the presence of high water content. The most abundant radical species were identified as DMPO–alkyl radical adducts. Besides monitoring the electron transfer during the free radical formation from lipid compounds, breakthroughs might also be achieved by detecting the changes among nonlipid compounds, such as the degradation of antioxidants (Jerzykiewicz et al., 2013). With the combination of the traditional and novel methods, such as EPR, an artificial neural network can be built to detect the free radicals generated in the kinetic model (Huang et al., 2021).

### 3.2 Comprehensive insights into lipid oxidation in W/O emulsions via oxidomics approach

Lipid oxidation in W/O emulsions is not only determined by the balance of oxidants and antioxidants but also by the physical factors. However, most of the studies are attempting to understand lipid oxidation mechanisms by applying a method to track a single chemical target (group). To better illustrate the mechanisms of lipid oxidation, the formation and degradation of some important minor compounds or microstructure changes require deeper investigations. Furthermore, there is a growing recognition of the importance of taking a multiomics approach to study lipid oxidation in W/O emulsions, incorporating chemical and physical factors, sensory qualities, and nutritional value, to gain a more complete understanding of this process. Hence, an oxidomics approach has been recently suggested for identifying novel markers in lipid oxidation and monitoring the dynamical lipid oxidation progress in a complex food system (Paradiso et al., 2018).

#### 3.2.1 Chemical markers for lipid oxidation in W/O emulsions based on oxidomics approach

In traditional research, omics studies are often performed by chromatographic methods, measuring many products simultaneously, allowing researchers to identify different lipid classes, such as oxidized and nonoxidized TAG, phospholipids, fatty acids, and cholesterols. In a recent study, an oxidomics approach was suggested to identify markers in the initial oxidation reactions in margarine, subjected to elevated temperatures (Grüneis et al., 2019). The oxidized lipids in margarine exposed to different heating conditions (25, 80, and 180°C, and 15, 30, 45, and 60 min) were analyzed by untargeted lipidomics using LC–MS. Interestingly, the epoxidation of TAG was found to be more abundant than hydroperoxidation during the early phase of lipid oxidation, whereas latter might occur via a hydrogen abstraction‐dependent pathway. Epoxides might be generated via a hydrogen abstraction‐independent pathway and can be considered as another lipid deterioration marker of lipid oxidation in W/O emulsions, specifically during the early stage of lipid deterioration. In another study, lipid oxidation was investigated in four commercial margarines stored at 4 and 15°C for 180 days by analyzing the comprehensive profile of peroxides, conjugated dienes, oxidized TAG, and volatiles (Fruehwirth, Egger, Flecker, et al., 2021). The formation of acetone during storage was proposed via the cyclization of peroxyl radicals followed by a reduction step and C–C and O–O bond cleavage. Additionally, the generation of acetone showed a close correlation to UFA, peroxide value, and conjugated dienes, thus suggesting a significant role of acetone in lipid oxidation during the storage of margarine. Oxidomics‐based lipid profiling can be useful during the lag phase of lipid oxidation, as it allows researchers to study the early molecular changes that occur before significant degradation of the lipid molecules takes place. It is also worth pointing out that besides lipid oxidation, co‐oxidation reactions between lipid compounds and proteins also occur in W/O emulsions with proteins as emulsifiers (Wazir et al., 2019). The health impact of these co‐oxidized products has been confirmed (Estévez & Xiong, 2019). Thus, more markers for lipid–protein co‐oxidation during the lag phase need to be identified by oxidomics, to predict and minimize the possible adverse effects on consumers’ health. This will provide crucial insights into the triggering mechanisms of lipid oxidation and the factors that drive its progression.

#### 3.2.2 Physicochemical markers of lipid oxidation in W/O emulsions based on oxidomics approach

A lot of classical chemical markers such as peroxide value and volatile components have been commonly used to evaluate the oxidative stability of W/O emulsions, and more novel chemical markers are still being discovered. However, very often critical micelle concentration (CMC) has been used as the only physical marker of initial lipid oxidation of W/O emulsions for decades (Brimberg, 1993; Garti, 2003). By applying oxidomics paradigm not only based on the chemical profile but also the microstructure changes, a study evaluated the role of FFA in the accretion of lipid oxidation in W/O emulsions (Paradiso et al., 2018). Interestingly, the addition of a variety of amounts of FFA, corresponding to the fatty acid composition in olive oil, to virgin olive oil did not change the micelle concentration. In the initial stage, the formation of hydroperoxides was accelerated only by lower concentrations (<5.0 mg/g) of FFA, whereas it was accelerated in the propagation stage in a dose‐dependent manner. FFA at a concentration below 5.0 mg/g were shown to act as the antagonist of TAG polymerization. As can be concluded from these results, the FFA content can be regarded as a marker for the initial lipid oxidation but also in propagation stage, prior to the CMC. This finding might call into question the sensitivity of this typical physical marker, CMC, in evaluating oxidative stability. As a consequence, the use of multiple physical markers could be necessary for providing a more comprehensive understanding of lipid oxidation and help to confirm or refine conclusions drawn from a single marker. There are many well‐established techniques to evaluate the physical stability of W/O emulsions, such as analyzing the lipid layer structure by EPR spectroscopy (Caianiello et al., 2020) and characterizing the droplet size and distribution by dynamic light scattering (Esposito et al., 2021) and transmission electron microscopy (Nguyen et al., 2015). In a very recent study, the application of mass spectrometric imaging accomplished the localization of emulsifiers on the surface of water droplets and structural determination of crystal network in W/O emulsions (Shiota, Shimomura, et al., 2018). Based on the dataset of physical properties, pseudophase diagrams (Golwala et al., 2020) or a coarse‐grained molecular model can be developed to predict the physical stability of W/O emulsions (Siddiqi & Herdes, 2022). These methods not only act as a powerful tool in revealing the interfacial behaviors but also offer the possibility of introducing interdisciplinary techniques in studying the lipid oxidation in W/O emulsions. Regrettably, the physical stability and oxidative stability of W/O emulsions have mostly been investigated separately, despite their interdependence. Hence, a great breakthrough can be made by combining the physical and chemical analysis to identify more novel lipid oxidation markers, since any exogenous conditions could alter microstructure and oxidant–antioxidant phenomena, thus activating different pathways of lipid oxidation. To the best of our knowledge, very few studies identified novel physical markers for lipid oxidation in W/O emulsions. By analyzing not only the traditional chemical markers for lipid oxidation such as peroxide value, anisidine value, and acid value but also the microstructure, rheological behavior, and viscoelasticity, it could be demonstrated that chemical as well as physical markers indicate lipid oxidation in W/O emulsions based on commercially refined oils (Chen et al., 2022). Studies traced the real‐time evolution of heat flux in W/O emulsions based on virgin plant oils by microcalorimetry (Dridi et al., 2017). According to the positive correlation between the enthalpy value and the conjugated diene hydroperoxide content, the enthalpy value can be used as a novel marker for real‐time monitoring of the lipid oxidation process in W/O emulsions, on a real‐time basis. While these approaches are convenient and reliable, the major drawback of these studies is the lack of comprehensive chemical analysis. In a further study, a correlation of the chemical profile and the thermal properties of 21 virgin olive oils was analyzed, confirming that thermal properties could be very useful markers for evaluating lipid oxidation in W/O emulsion (Chiavaro et al., 2013). By using an oxidomics approach, it may be possible to identify physical–chemical markers that can indicate lipid oxidation in W/O emulsions and to provide insights into the mechanism of lipid oxidation during the lag phase, storage, and processing.

#### 3.2.3 Multidimensional network based on oxidomics integrating chemical, physical, sensory, and nutritional dimensions

The core principle of research about lipid oxidation is to benefit the consumers, because the oxidized products may impair the sensory quality and nutritional value, and even cause a health impact. A study found correlations among sensory‐active components, photosensitizers, volatile components, peroxides, and TBARS in light‐exposed butter samples packed in air or nitrogen (Veberg et al., 2007). The degradation of photosensitizers was observed at the initial stage of lipid oxidation and was highly related to the sensory quality showing an enhanced release of acidic and rancid flavors. Though promising possibilities in predicting the sensory quality by novel markers can be seen in this study, the evidence of the correlation between various indicators of lipid oxidation and sensory quality was provided, further highlighting the importance of integrating experimental data from physical stability, oxidative stability, and sensory quality. We here suggest setting up a comprehensive multidimensional network considering chemical, physical, sensory, and even nutritional dimensions by oxidomics (Table 3). It is also worth mentioning that the oxidation of W/O food emulsions continues during digestion. The oxidized lipids formed under digestive conditions might be absorbed by the gastrointestinal tract; hence, the oxidation markers formed during digestion, the possible molecular pathways, and their health effects are also of great concern. In light of this, understanding the oxidation process and its effects during digestion is crucial in evaluating the overall impact of W/O food emulsions on human health and wellness. Further findings could be expected by using the oxidomics approach as well as by applying correlation analysis between physical datasets, chemical profiles, and biological endpoints, to benefit the prediction of the sensory quality and the nutritional value of the W/O food emulsions before they enter the market.

**TABLE 3:** Representative novel markers for lipid oxidation in water‐in‐oil (W/O) food emulsions based on oxidomics approaches.

W/O emulsions	Markers (reference)	Major techniques	Data integration
Margarine	Epoxides (Grüneis et al., 2019)	LC–MS; LC–MS/MS; GC–FID	XCMS online
	Acetone (Fruehwirth, Egger et al., 2021)	GC–FID; LC–MS/MS; DGF method C‐VI 6; Rancimat Method	Spearman's rank‐order correlation analysis
Virgin olive oil	Free fatty acid (Paradiso et al., 2018)	7,7,8,8‐Tetracyanoquino‐dimethane (TCNQ) solubilization technique; SAXS; HS‐SPME–GC–MS; HPSEC; HPLC–ESI–MS	Principal components analysis (PCA); regression analysis
W/O emulsion based on refined commercial oils	Viscoelasticity (Chen et al., 2022)	CLSM; PLM; rheometer; POV; anisidine value; acid value	
Emulsions based on virgin plant oil	Enthalpy value (Dridi et al., 2017)	Microcalorimetry	Correlation analysis
Virgin olive oils	DSC profile (Chiavaro et al., 2013)	GC–FID; HPLC–DAD; HPSEC; DSC	PCA; correlation analysis
Butter	β‐Carotene (Veberg et al., 2007)	GC–MS; fluorescence spectroscopy; sensory evaluation; TBARS; POV	Partial least squares (PLS) regression; multivariate curve resolution (MCR)

Overall, the application of the oxidomics approach provides a holistic view of lipid oxidation and allows the discovery of novel oxidation markers, thereby describing possible molecular pathways and supramolecular interactions underlying lipid oxidation in W/O emulsions needed to develop targeted protective approaches. However, there are also limitations to oxidomics. For example, the complexity of lipid oxidation processes can make it difficult to accurately interpret the results, and the multiple steps involved in omics technologies can introduce errors or biases. Additionally, oxidomics often requires large amounts of sample material and specialized equipment, making it a challenging and resource‐intensive approach. By focusing on improving the accuracy and reliability of oxidomics through the use of advanced technologies, innovative and standardized analytical methods and guidelines, and efficient sample preparation techniques, future researchers can overcome the limitation of oxidomics and gain a deeper understanding of lipid oxidation in W/O food emulsions, benefiting both consumers and the food industry.

## 4 TARGETED PROTECTIVE APPROACHES TO COMBAT LIPID OXIDATION IN W/O FOOD EMULSIONS

Plenty of attempts have been made to extend the shelf life of emulsion systems by keeping the physical stability and slowing down the lipid oxidation process. Regarding the complexity of physical and chemical changes, new mechanisms are being revealed by applying the novel techniques. Therefore, considering the affecting factors and possible mechanisms of lipid oxidation, targeted approaches can be proposed to protect the W/O emulsions from lipid oxidation by optimizing the chemical composition of both phases, stabilizing the microstructure, or reducing the effects of exogenous prooxidant factors.

### 4.1 Optimization of the composition of the lipid phase

As the major components in W/O emulsions, the composition of the lipid phase not only determines the oxidative stability but also affects the sensory property and nutritional value. So, to improve the oxidative stability of W/O emulsion by optimizing the composition of the lipid phase, not only chemical properties, such as the degree of unsaturation, but also the texture and microstructure should be considered.

The solubility and diffusion of oxygen in W/O emulsions can be diminished by higher viscosity of the lipid phase (Chaix et al., 2014; Pénicaud et al., 2010). Hence, the amounts of saturated fatty acids or components with higher viscosity can be elevated to increase the content of crystalline lipids without compromising the sensory qualities and nutritional value of W/O food emulsions (Table 4). In a study about iron‐fortified W/O emulsion, the emulsion with oil phase based on rice bran stearin contained 35.5% saturated fatty acids and showed stronger oxidative stability than the emulsion prepared with rice bran oil containing 23.8% saturated fatty acids, which can be suggesting to the formation of a stronger lipid crystal networks in the former emulsion (Prichapan et al., 2018).

**TABLE 4:** Representative modified composition of lipid phase to prevent lipid oxidation in water‐in‐oil (W/O) food emulsions.

W/O emulsions	Alternatives (reference)	Original fatty acid composition
Encapsulation of iron in W/O emulsion	Replacement with rice bran stearin (contains 35.5% saturated fatty acids) (Prichapan et al., 2018)	Rice bran oil (contains 23.6% saturated fatty acids)
Margarine	Partial replacement by duck oil (contains 48.70% oleic acid) (Shin et al., 2021)	Hydrogenated soybean oil (contains 85.6% stearic acid)
	Supplement with 5%, 10%, 15%, and 20% chia oil (Nadeem et al., 2017)	Nonhydrogenated palm oil, palm kernel, and butter
Margarine/ice cream	Replacement by enzymatic interesterified *Moringa oleifera* seed oil (oleic acid content >73%) (Dollah et al., 2016)	Commercial formulation
Margarine	Replacement by vitamin C (VC)‐loaded oleogel with corn oil and monoglyceride stearate (Wang, Wang, et al., 2021)	Commercial formulation

In the case of margarine, vegetable oils are commonly used to form the lipid phase. Hydrogenation of these vegetable oils is an alternative way to improve the texture of margarine. However, *trans* fatty acids can be generated during hydrogenation, which may cause harmful health effects. Hence, compared to the hydrogenation of the vegetable oils, optimizing the fatty acid composition of the lipid phase in margarine with lipids facilitating soft texture might lead to higher oxidative stability and health benefits as well. For example, the replacement of hydrogenated soybean oil by duck oil promoted more β form of fat crystallization, thus increasing the viscosity of margarine and providing a smoother texture, leading to a prolonged shelf life and importantly, improved better sensory qualities (Shin et al., 2021). In another study, the optimization of the fatty acid composition of the margarine by supplementing with an oil prepared from chia seeds also caused an enrichment of the margarine with polyphenols from the seeds (Nadeem et al., 2017). The margarine supplemented with chia oil showed a higher content of *ω*−3 fatty acids, better oxidative stability due to the phenolic compounds in chia oil, and an uncompromised sensory quality. To avoid the formation of *trans* fatty acids, another preferable approach is using lipase‐catalyzed enzymatic interesterification instead of hydrogenation, which can keep the special texture of the lipid phase in margarine. An enzymatic interesterified *Moringa oleifera* seed oil, which contained a high amount of oleic acid and a very low amount of PUFA, was applied in margarine (Dollah et al., 2016). Soft margarine and ice cream formulated from enzymatic interesterified *M. oleifera* seed oil showed to have a better oxidative stability during storage. It is also worth mentioning that along with the optimization of the fatty acid composition in the oil phase of margarine, the development of oleogel‐based margarine is growing with the popularity of healthy diets and functional foods. The replacement of the entire lipid phase of margarine by vitamin C‐loaded oleogel with corn oil and monoglyceride stearate was a preliminary attempt in developing a functional margarine (Wang, Wang, et al., 2021). Despite some differences that may exist regarding the appearance and texture of oleogel‐based and commercial margarine, the oxidative stability got significantly improved due to the enrichment with vitamin C as a hydrophilic antioxidant. Thus, to improve oxidative stability by optimizing the lipid phase, further studies can focus on modifying the fatty acid composition in the lipid phase by replacing the lipid phase with oxidatively stable oleogel structures.

### 4.2 Modification of the encapsulated formulation

Transition metals can act as nutritional supplements added to the aqueous droplets but also catalyze the lipid oxidation in W/O emulsions. However, their prooxidant effect can be weakened by strongly bounded ligands. For example, a study demonstrated that after enrichment of aqueous droplets with ferrous ions, the rates of lipid oxidation in W/O emulsions are as follows: ferrous chloride > ferrous sulfate > ferrous lactate > ferrous gluconate (Dridi et al., 2016). Hence, in artificial W/O emulsions delivering transition metals, the selection of proper ligands may lower lipid oxidation by using hydrophilic chelating agents (e.g., proteins, gluconate, lactate).

### 4.3 Customization of processing parameters

Applying suitable processing parameters can be one of the key factors for obtaining W/O emulsions with prolonged shelf life. Due to the Ostwald ripening, coalescence is unavoidable during the storage of W/O emulsions. To improve the physical stability and texture, producers are designing W/O emulsions with a smaller water droplet size. Smaller water droplets in margarine were observed at 10°C rather than at 30°C (Shiota, Kamigaki, et al., 2018), suggesting that in industrial processing, fat crystallization is closely related to temperature control. Utilization of a faster cooling rate (5°C/min) led to a smaller water droplet size and longer physical stability of the fat crystal network than a slower cooling rate (1°C/min) and a bigger temperature cooling range (from 45°C to either 25°C or 4°C) (Ghosh et al., 2015). In addition, the application of the ultrasonic technique in the processing of W/O emulsion is also an effective way to reduce the water droplet size and inhibit the sedimentation, thus improving the physical stability (Al‐Maqtari et al., 2021). However, these methods only considered the physical stability of W/O emulsions without evaluating the oxidative stability. W/O emulsions may be prepared by high‐shear and high‐pressure homogenization of water and oil phases. A long‐term oxidative stability can be achieved by avoiding the interaction with prooxidants during the processing of W/O food emulsions. For example, during the processing, some transition metals may be transferred from the metal steel propeller into the emulsion. Sufficient stirring can also lead to oxygen enrichment in the emulsion system. Hence, avoiding interaction with oxygen and transition metals during the preparation of emulsions needs to be emphasized, such as by applying the plastic propeller and replacing the oxygen by nitrogen in the stirring step. Recently, a polydimethylsiloxane‐based microfluidic device was developed to provide a microscale method for forming W/O emulsion with small droplet size and polydispersity. This device not only improved the physical stability by reducing the water droplet size, but also accomplished the formation of W/O emulsions without prehomogenization, thereby avoiding the contact with air and improving the oxidative stability (Nieves et al., 2021).

In addition to the lack of consideration of both chemical and physical stability, lots of applications of novel techniques remain at the theoretical and laboratory level and have not yet achieved industrial production. To establish industrial production methods of W/O emulsions, the processes need to be further optimized to improve the cost efficiency, thus achieving mass production.

### 4.4 Application of novel stabilizing methods

The physical and chemical interaction at the interface determines the structural and oxidative stability of W/O emulsions (Nesterenko et al., 2014). Hence, to delay the rate of lipid oxidation in W/O emulsions, a wide range of novel emulsifiers and particles have been developed to act as stabilizers (Table 5).

**TABLE 5:** Representative stabilizing methods to limit lipid oxidation in water‐in‐oil (W/O) food emulsions.

Preferable stabilizing methods	Stabilizers (reference)
Designing cosurfactants with appropriate concentrations and ratio	0.5% diacylglycerols (DAG) + 0.5% polyglycerol polyricinoleate (PGPR) (Yang et al., 2020) 6 wt% DAG + 1 wt% PGPR (Liu et al., 2022) PGPR + lecithin (Okuro et al., 2019) Cellulose nanofibrils + SDS (Huan et al., 2017)
Combination of surfactants and nanoparticles	Polymeric surfactant + silica nanoparticles (Fernández et al., 2015)
Choosing natural particles to form Pickering W/O emulsions	Tea polyphenol palmitate (Luo et al., 2019) Luteolin (Wang, Lu, et al., 2021)
Application of surface‐active biopolymers	Octenyl succinate starch; corn starch (CS), octenyl succinic anhydride‐modified corn starch (Gao et al., 2021) Eggshell membrane protein hydrolysates + culled banana resistant starch (Jain & Anal, 2018) Whey protein isolate (Yi et al., 2014)

The effectiveness of some individual emulsifiers for reducing the interfacial tension and stabilizing the fat crystal network has been confirmed. So, more researchers are exploring the effects of combined emulsifiers on the W/O emulsion properties. The combination of 0.5% of DAG with 0.5% of PGPR as co‐emulsifier of a W/O emulsion based on canola oil and 20% water decreased the waterdrop size by the formation of the Pickering crystal shell at the surface, thus improving the physical stability under freeze–thaw conditions (Yang et al., 2020). The co‐stabilizing effects of DAG (6 wt%) and PGPR (1 wt%) were further confirmed in a W/O emulsion with 80% water content (Liu et al., 2022). Improved stabilizing effects were also observed in a sunflower oil‐based W/O emulsion co‐emulsified by PGPR and lecithin (Okuro et al., 2019) and cellulose nanofibrils and SDS (Huan et al., 2017). The combination of emulsifiers achieves better physical stability of W/O emulsions that might also contribute better oxidative stability. However, the possible contact between emulsifiers and prooxidants is often overlooked by researchers, which may affect the rate of lipid oxidation since they might facilitate the transfer of electrons (Laguerre et al., 2017). In addition, because of the difficulty of stabilizing all droplets with different sizes by only emulsifiers, particles are proposed to be used to stabilize bigger water droplets and form a thicker barrier between the oil and water phases, which hinder the interaction of prooxidant and lipids (Zhao et al., 2013), while surfactant molecules can be used to stabilize small droplets (Drelich et al., 2010; Nesterenko et al., 2014). W/O emulsions based on corn oil stabilized by a polymeric surfactant (87%−89% hydrolyzed proteose medium and 0.1 wt% polyvinyl alcohol) and solid silica nanoparticles showed long‐term storage stability and biological harmlessness (Fernández et al., 2015).

Although these commonly used emulsifiers or particles are considered safe, with the recently expanding attentiveness to nutritional value and safety of W/O emulsions foods, looking for natural alternatives of stabilizers with self‐emulsifying and antioxidative properties is becoming a research trend. Natural particles are better alternatives to form Pickering W/O emulsions, due to their biocompatibility and antioxidant activity. The addition of tea polyphenol palmitate particles into camellia oil formed a stable fat crystal network in the W/O emulsion with high storage, thermal, and freeze–thaw stability (Luo et al., 2019). The oxidative stability of pine nut oil‐based W/O emulsions was improved under UV exposure, high temperature, high NaCl concentrations, and simulated gastrointestinal conditions by the addition of luteolin micro/nanoparticles as a stabilizer (Wang, Lu, et al., 2021).

Moreover, surface‐active biopolymers with antioxidant effects, high biodegradability, and high biocompatibility are designed, validated, and applied in W/O emulsions. Corn starch and octenyl succinic anhydride‐modified corn starch were confirmed to form a stable fat crystal network in W/O emulsion based on soybean oil by amylose hydrogen bonding, leading to high stability during simulated gastrointestinal digestion (Gao et al., 2021). A combination of eggshell membrane protein hydrolysates and culled banana resistant starch showed synergistic effects by increasing physical stability and decreasing the lipid oxidation rate in W/O emulsions (Jain & Anal, 2018). The stabilizing effects of electrically charged emulsifiers are based on an electrostatic mechanism, causing a possible instability when pH is changed. For example, a stronger oxidative stability was observed in the W/O emulsion prepared with WPI at pH 7.0 than at pH 3.0, which can be attributed to a more pronounced chelation of transition metals and scavenging of free radicals (Yi et al., 2014). Furthermore, the combination of WPI with SDS increased the inhibiting effects on lipid oxidation in the W/O emulsion due to the protein‐denaturation effect of SDS (Yi et al., 2014), which exposes the amino acids from the core of proteins (Parker & Song, 1992).

Overall, increasing the physical and chemical stability of W/O emulsion by using food‐derived stabilizers has been the developmental trend in recent years. Especially, the mixed‐particle/surfactant stabilizing system showed synergistic effects, which may therefore lead to stable W/O emulsions with higher physical and chemical stability. Thus, to prevent lipid oxidation and improve the physical stability of W/O emulsions, the optimization of stabilizers should be put into practice by using natural emulsifiers with bioactivities, particularly, antioxidant activity. Moreover, considerable progress should also be made to study the proper concentrations, ratios of these co‐stabilizers, and some other characterizing parameters such as pH value. Molecular modification can be achieved in bioemulsifiers to gain bioactivities or even unique flavors. Physical modification can be done in colloidal particles to obtain improved stabilities in combination with emulsifiers or antioxidants.

### 4.5 Addition of appropriate antioxidants

In the traditional food industry, chemosynthetic compounds such as butylated hydroxytoluene (BHT), butylated hydroxyanisole, and ethylenediaminetetraacetic acid are commonly used as antioxidants to prevent lipid oxidation in emulsions (Gorji et al., 2016). Nevertheless, the toxicity and carcinogenicity caused by these compounds are inevitable, especially when the concentration reaches a threshold value (Martínez‐Tomé et al., 2001). Therefore, the application of natural antioxidants to replace chemosynthetic additives is a growing trend in the food industry, since more consumers can be appealed by the health effects of natural products (Table 6).

**TABLE 6:** Representative antioxidants used for decreasing lipid oxidation in water‐in‐oil (W/O) food emulsions.

Sources of antioxidants	Antioxidants (reference)
Chemosynthetic compounds	Butylated hydroxytoluene, butylated hydroxyanisole, and ethylenediaminetetraacetic acid (Gorji et al., 2016)
Minor compounds from W/O emulsions	Phytosterols (Sopelana et al., 2016) Carotenoids and β‐carotene (Kalogeropoulos & Tsimidou, 2014; Young & Lowe, 2018) Polyphenols (Zeb, 2021)
Edible plant extracts	Tea extract (Chen, Rao, et al., 2016) Green tea polyphenols (Fruehwirth, Egger, Kurzbach, et al., 2021) Rosemary extract (Engler Ribeiro et al., 2017)
Food processing byproducts	Pecan nut shell extract (Engler Ribeiro et al., 2017) *Opuntia ficus‐indica* peel extract (Chougui et al., 2015) Pomegranate peel and pomegranate seed extracts (Çam et al., 2013)
Phenolipids	β‐Sitosteryl sinapate, β‐sitosteryl caffeate, and β‐sitosteryl ferulate (Rabiej‐Kozioł et al., 2020)

Some minor compounds in W/O emulsions have been confirmed to act as antioxidants. For example, during the heating process of margarine at 180°C for 240 min, phytosterols were confirmed to delay the formation of lipid oxidation products (Sopelana et al., 2016). Some pigments, especially carotenoids and β‐carotene, can easily react with free radicals, thereby acting as antioxidants (Kalogeropoulos & Tsimidou, 2014; Young & Lowe, 2018). According to a lipid oxidation monitoring study in a W/O emulsion system, carotenoids and β‐carotene delayed the initial oxidation of lipids in the system (Sattar et al., 1976). In addition, polyphenols present in natural oils are also an excellent source of antioxidants (Zeb, 2021). The enrichment with these antioxidants in appropriate concentrations might be an effective solution.

It is noteworthy to consider the possibility of applying antioxidant peptides and protein hydrolysates, given their ability to act as electron donors due to the presence of amino acids with reactive functional groups, such as thiols and phenols, to prevent lipid oxidation in W/O emulsions. However, it is necessary to optimize the formulation of W/O emulsions using peptides and protein hydrolysates as antioxidants due to their emulsifying properties. In addition, the production process for protein hydrolysates is generally complex and time‐consuming, and the production cost of peptides is even higher compared to protein hydrolysates due to the complex and labor‐intensive process of synthesizing peptides. Hence, easily available raw materials are preferred by researchers and food industries as the source of antioxidants. Recently, the addition of edible plant extracts as natural antioxidants has been widely used in the processing of food emulsions, which benefit from the large amounts of polyphenols in plants (Choe, 2020). For example, tea extract, commonly used as an edible source of antioxidants, significantly protected a W/O emulsion from lipid oxidation, which may be due to the free radical scavenging and metal chelating activity of polyphenols (Chen, Rao, et al., 2016). A recent study also confirmed that polyphenols from green tea at low concentrations decreased lipid oxidation in margarine by increasing the metal chelation at the interface (Fruehwirth, Egger, Kurzbach, et al., 2021). Furthermore, using the byproducts from food processing as a source of antioxidants is gaining acceptance as a means of reducing food waste. Compared to BHT, the extracts of pecan nut shell and rosemary showed similar effects on preventing lipid oxidation of margarine, suggesting the possibility of using these extracts as alternatives to synthetic antioxidants (Engler Ribeiro et al., 2017). Another study reported the enrichment with byproducts from *Opuntia ficus‐indica* peel showing higher oxidative stability in margarine compared to addition of vitamin E (Chougui et al., 2015). The utilization of pomegranate byproducts (pomegranate peel and pomegranate seed extracts) inhibited the lipid oxidation in ice cream without compromising sensory properties (Çam et al., 2013). Synergistic effects in antioxidant activity could be observed in W/O emulsions with protein–antioxidant adducts (Almajano & Gordon, 2004; Conde et al., 2011). Hence, plant‐based products can be a good resource of natural antioxidants for inhibiting lipid oxidation as well as providing W/O emulsions an improved nutritional value.

Regarding the appropriate use of antioxidants, the location of antioxidants in the W/O emulsions has to be taken into consideration since it also affects the oxidative stability of emulsions as explained by the polar paradox (see Section 2.2.3). Hydrophobic antioxidants, although more soluble in the oil phase due to their low polarity, may be less effective than hydrophilic antioxidants at the water–oil interface, which is the main site of lipid oxidation in W/O emulsions. However, hydrophilic antioxidants, such as ascorbic acid and phenolic compounds, are being carried in the water droplets, and thus may also provide limited protection due to their limited access to the lipid phase. To enhance the functionality of hydrophilic antioxidants at the interface, small molecular modifications enable the change of the polarity of these hydrophilic antioxidants, thus allowing them to function in the lipid phase as well. The esterification of sterols with phenolic acids can increase the utilization of hydrophilic antioxidants in the lipid phase (Laguerre et al., 2015). A study demonstrated the antioxidant effects of steryl hydroxycinnamates synthesized from caffeic acid, ferulic acid, and sinapic acid in margarine (Rabiej‐Kozioł et al., 2020). Designing targeted antioxidants, such as phenolipids, by a proper molecular modification based on the cutoff effect (Laguerre et al., 2009, 2010) to adjust their exact location can be a strategy to prevent lipid oxidation in W/O food emulsions.

Present studies are focusing on the safety, efficiency, and nutritional value of antioxidants to prevent lipid oxidation in W/O food emulsions. Notably, before taking the laboratory theory results into the practice of industrial application, the following considerations should be addressed:

1. The activity of antioxidants can be affected by pH or temperature; thus, pH/temperature‐stable antioxidants should be developed by screening natural products or strategic molecular modification;
2. The synergistic effects induced by the combination of the appropriate emulsifiers and antioxidants should be considered due to the effects of the emulsifiers on the diffusion of antioxidants at the water–oil interface;
3. Plant extracts may contain many bitter substances. Their unpleasant taste has to be evaluated or diminished, for example, by molecular modifications.

### 4.6 Designing antioxidant packaging materials

Oxygen can be considered one of the most important factors in the surrounding environment of regulating lipid oxidation of W/O food emulsions. So, it is essential to build an effective physical barrier between the food and the atmosphere. Considering the headspace between the package and food products, packing under vacuum or nitrogen (Coupland & McClements, 1996), and application of packaging materials with low oxygen permeability produced by nanotechnology (Duncan, 2011) to decrease the chance of oxygen diffusion in W/O emulsions are the most common strategies in the traditional food industry. Although it is difficult or even impossible to achieve a complete elimination of oxygen as well as moisture from the atmosphere, plenty of packaging materials have been designed to maximize their protection toward lipid oxidation in W/O food emulsions (Table 7).

**TABLE 7:** Representative functional packaging used for inhibiting lipid oxidation in water‐in‐oil (W/O) emulsions.

Functions of packaging	Packaging materials (reference)
Antioxidative activity	Tocopherol, ascorbic acid, gallic acid, and biobased multilayer film (Pant et al., 2017) Ferulic acid and molecularly imprinted hydrogels (Benito‐Peña et al., 2016) Selenium nanoparticles (Vera et al., 2018)
UV and oxygen barrier	Cellulose nanocrystal and chitosan (Yadav et al., 2020)
Intelligent packaging	Polylactic acid/titanium dioxide/lycopene nanocomposite film (Pirsa & Asadi, 2021)
	Pectin/nanoclay/*Carum copticum* essential oils/β‐carotene (Asdagh & Pirsa, 2020)

Adding active antioxidants into the packaging has been suggested as a promising strategy to delay the lipid oxidation of food emulsions. In particular, natural antioxidants from plants such as tocopherol, ascorbic acid, and gallic acid have been incorporated into the packaging to extend the shelf life of food products (Pant et al., 2017). With the enrichment of molecularly imprinted hydrogels with ferulic acid, an antioxidant packaging material was designed for butter, which protected it better from lipid oxidation than the packaging without antioxidants (Benito‐Peña et al., 2016). A new antioxidant multilayer packaging based on selenium nanoparticles has been developed and demonstrated to inhibit the lipid oxidation of PUFA (Vera et al., 2018).

Although using packaging material capable of decreasing the level of oxygen can be an effective way to protect from lipid oxidation, it is unavoidable to use transparent packaging in food retail. In this regard, the application of transparent packaging materials that can act as both UV and oxygen barriers is urgently needed. The combination of biopolymers and nanocomposite, such as cellulose nanocrystal and chitosan (Yadav et al., 2020), has been experimentally confirmed to protect food from UV and oxygen.

More importantly, when W/O food emulsions arrive at the supermarket, the manufacturers are unable to further control the lipid oxidation process. Hence, offering guidelines to consumers via packaging to attract their attention to lipid oxidation can be a promising approach to prevent the health impact caused by lipid oxidation products. For instance, a polylactic acid/titanium dioxide/lycopene nanocomposite film has been designed and used for protecting margarine from the lipid oxidation (Pirsa & Asadi, 2021). Interestingly, the color of the film changed over the storage duration, which was related to the degree of lipid oxidation. Another packaging film based on pectin/nanoclay/*Carum copticum* essential oils/β‐carotene was developed to prevent lipid oxidation in butter. The color of the film acts as a β‐carotene‐mediated indicator for lipid oxidation (Asdagh & Pirsa, 2020). These antioxidant packaging can intelligently determine the degree of oxidation in W/O emulsions, and act as a sensor of the actual expiration date under different storage conditions.

The addition of antioxidants as oxygen scavengers and color‐changing compounds in packaging materials inhibits lipid oxidation of W/O food emulsions as well as provides indicative signals to consumers about the food quality. However, more efforts should be made to reduce the producing cost, reduce manufacturing time of these intelligent packaging, and achieve a higher commercial availability. In addition, future studies should also take the protecting effects of temperature changes into account, because a temperature change might affect the physical and oxidative stability of W/O food emulsions.

Despite it being difficult to completely inhibit lipid oxidation of W/O emulsions, more novel controlling approaches are still needed to minimize the lipid oxidation in W/O food emulsions. Targeted protective approaches should be proposed regarding the specificities of different W/O food emulsions, which may be based on the markers identified by oxidomics. Among all strategies to enhance oxidative stability, a core principle should adhere to consumers’ requirements for sensory qualities and nutritional value. Hence, the characterization of sensory qualities and investigation of bioactivities are essential in the evaluation of the lipid oxidation‐preventing effects of a novel or optimized W/O food emulsion.

## 5 CONCLUSION

In the current comprehensive review, we discussed the factors that may affect the oxidation process, highlighted the possible mechanisms of lipid oxidation in W/O emulsions from both chemical and physical dimensions, and suggested oxidomics to better understand the molecular and supramolecular mechanisms and identify oxidation markers, allowing to implement targeted protective approaches to decrease the lipid oxidation rate in W/O food emulsions.

**FIGURE 5:**
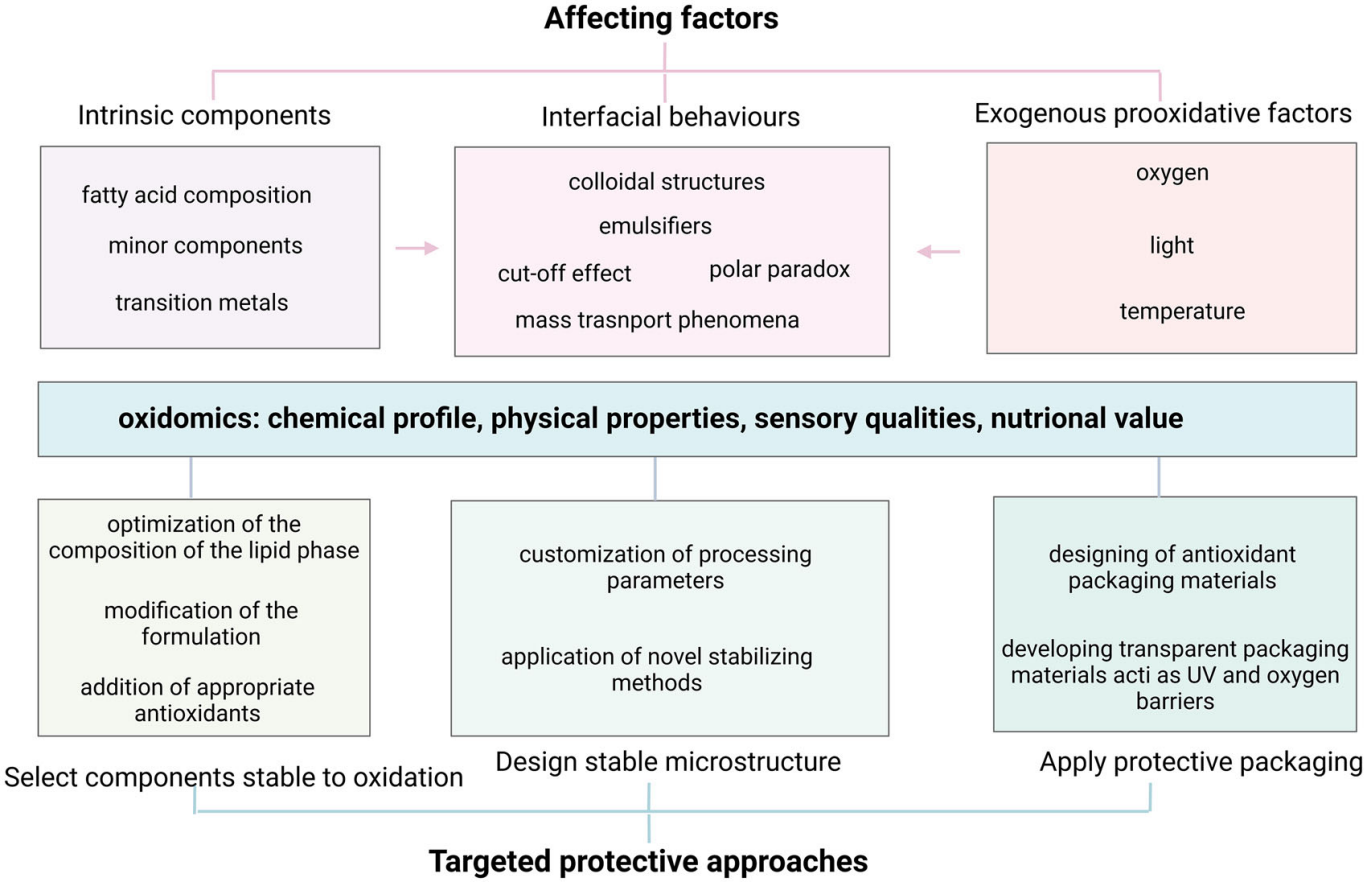
The key influential factors in lipid oxidation of water‐in‐oil (W/O) emulsions and the targeted protective approaches.

The lipid oxidation in W/O emulsions is based on radical chain reactions and can be influenced by (1) intrinsic components such as the composition of lipid phases, transition metals, and minor compounds; (2) physical properties such as colloidal structures, water droplet size and distribution, and interfacial tension, which can be mediated by emulsifiers; and (3) exogenous prooxidative factors such as oxygen concentration, light, and temperature. Therefore, studying the complex mechanisms of lipid oxidation by commonly used oxidative stability indices is not sufficient. Moreover, as the main reaction site for lipid oxidation, more attention should be paid to the chemical and physical changes during the oxidation process at the water–lipid interface. Few existing studies suggest a combination of the analyses of physical properties and chemical profiles to interpret the mechanisms of lipid oxidation in W/O emulsions. Hence, we are proposing the novel oxidomics approach that can be applied in future studies to detect relevant and specific markers of lipid oxidation by combining multiple analytical techniques and correlation analysis. Especially the association of chemical and physical markers with sensory properties would be an asset. Plenty of attempts have been made to prevent lipid oxidation in W/O emulsion, such as altering the chemical composition of the lipid phase, optimizing the processing parameters, choosing appropriate stabilizers, adding antioxidants, and developing antioxidative packaging. To achieve synergistic effects of these approaches, it is of great significance to combine the analyses of lipid oxidation mechanisms, physical properties, sensory qualities, and nutritional value, allowing to design the targeting protective approaches (Figure 5).

**FIGURE 6:**
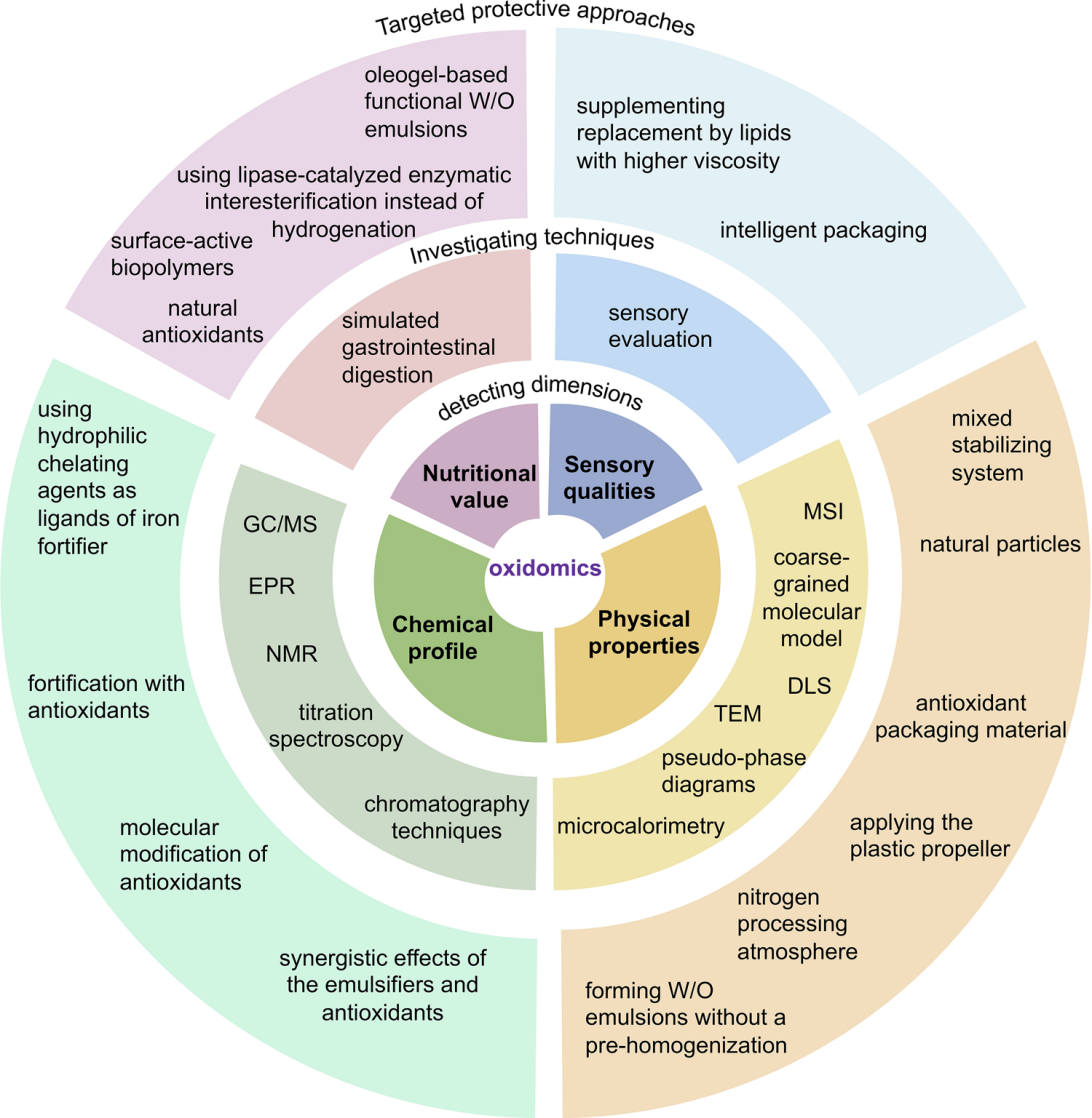
Outlook of future academic research and industrial improvement.

In general, considering consumers’ concerns about the loss of nutritive value, limited shelf life, and the long‐term health impact of lipid oxidation produces in W/O food emulsions, revealing the lipid oxidation mechanisms in W/O emulsion by novel techniques and applying targeted protective approaches is urgently needed. The comprehensive analysis proposed oxidomics as a promising strategy to obtain a holistic view necessary for achieving a breakthrough in the studies about lipid oxidation and the development of W/O food emulsions with improved stability (Figure 6).

## AUTHOR CONTRIBUTIONS

**Yifan Bao**: Writing – original draft; visualization; conceptualization. **Marc Pignitter**: Funding acquisition; writing – review and editing; supervision; conceptualization.

## CONFLICT OF INTEREST STATEMENT

The authors declare no conflicts of interest.
